# Adipose Tissue-Derived Mesenchymal Stem Cells as a Potential Restorative Treatment for Cartilage Defects: A PRISMA Review and Meta-Analysis

**DOI:** 10.3390/ph14121280

**Published:** 2021-12-08

**Authors:** Henry Yue-Hong Meng, Victor Lu, Wasim Khan

**Affiliations:** 1 Faculty of Medicine, The Chinese University of Hong Kong, Central Ave., Hong Kong 999077, China; hyhmeng@gmail.com; 2 School of Clinical Medicine, University of Cambridge, Cambridge CB2 0SP, UK; victorluwawa@yahoo.com.hk; 3 Division of Trauma & Orthopaedic Surgery, Addenbrooke’s Hospital, University of Cambridge, Cambridge CB2 0QQ, UK

**Keywords:** adipose tissue, cartilage repair, infrapatellar fat pad, mesenchymal stem cells, osteoarthritis, transplantation

## Abstract

Cartilage defects are a predisposing factor for osteoarthritis. Conventional therapies are mostly palliative and there is an interest in developing newer therapies that target the disease’s progression. Mesenchymal stem cells (MSCs) have been suggested as a promising therapy to restore hyaline cartilage to cartilage defects, though the optimal cell source has remained under investigation. A PRISMA systematic review was conducted utilising five databases (MEDLINE, EMBASE, Cochrane Library, Scopus, Web of Science) which identified nineteen human studies that used adipose tissue-derived MSC (AMSC)-based therapies, including culture-expanded AMSCs and stromal vascular fraction, to treat cartilage defects. Clinical, imaging and histological outcomes, as well as other relevant details pertaining to cartilage regeneration, were extracted from each study. Pooled analysis revealed a significant improvement in WOMAC scores (mean difference: −25.52; 95%CI (−30.93, −20.10); *p* < 0.001), VAS scores (mean difference: −3.30; 95%CI (−3.72, −2.89); *p* < 0.001), KOOS scores and end point MOCART score (mean: 68.12; 95%CI (62.18, 74.05)), thus showing improvement. The studies in this review demonstrate the safety and efficacy of AMSC-based therapies for cartilage defects. Establishing standardised methods for MSC extraction and delivery, and performing studies with long follow-up should enable future high-quality research to provide the evidence needed to bring AMSC-based therapies into the market.

## 1. Introduction

Cartilage, an important element of synovial joints, is composed of chondrocytes that lay down a highly organised extracellular matrix (ECM), consisting of water, type II collagen, glycosaminoglycans (GAGs) and proteoglycans, amalgamated into a dense collagenous network, which is responsible for its unique mechanical properties [1]. As well as crucial shock-absorbing and gliding properties that cartilage is well known for, cartilage ECM also has a role in chondrocyte homeostasis and cartilage phenotypic stability via chemokine signalling [2], and the synthesis of functional components of the skeletal system during embryogenesis [3]. Hence, any insults to cartilage integrity and the surrounding ECM will have a profound impact on chondrocytes, which in turn will lead to ECM composition changes, all in a vicious cycle [4].

Cartilage defect is a risk factor for osteoarthritis, and current therapies aim to relieve symptoms or prevent further degenerative changes to the articular cartilage. Cartilage damage is a key feature in degenerative diseases such as osteoarthritis (OA), with 26.6% of those aged 45 or above having a diagnosis of OA, which is projected to increase in the near future [5]. The World Health Organisation suggested that 25% of OA patients are unable to perform major day-to-day activities and 80% suffer from movement limitations [6]. Furthermore, knee and hip OA patients are at a higher risk of suffering from depression [7] and 1.27 times more likely to have suicidal thoughts [8]. With such widespread global impact and causing a significant socioeconomic burden for the patient, it is of great interest to scientists to find a solution for this.

Articular cartilage is avascular and devoid of lymphatics and nerves, limiting regenerative capabilities. Since the time of Hippocrates, many have astutely observed that once damaged, cartilage does not heal [9]. Hence, current treatments for early to moderate OA are mostly palliative such as weight reduction, more exercise, walking aids and thermal modalities [10]. Pharmacological treatment includes the administration of analgesics, non-steroidal, anti-inflammatory drugs and chondroprotective agents such as glucosamine sulphate, hyaluronic acid, chondroitin, growth factors and, hormones [11,12]. These management options are insufficient in those with severe OA. Since there has not been any approved Disease-Modifying Osteoarthritis Drugs (DMOAD), these patients require surgical interventions such as arthroscopic lavage and debridement to remove inflammatory mediators and loose cartilaginous debris [13], osteotomy and knee arthroplasty to retain function, all of which are costly, fraught with perioperative and post-operative complications, and sometimes lead to unsatisfactory outcomes [14].

Newer treatments aim to restore function by inducing cartilage regeneration. Studies aiming to stimulate cartilage formation have conducted trials on microfracture, a single-stage arthroscopic procedure, as a means for treating small-sized cartilage lesions. The purpose is to create fibrin clots to stimulate mesenchymal stem cell differentiation. However, the cartilage formed is fibrocartilage, which is inferior to normal hyaline cartilage. This could perhaps explain why clinical results have been mixed to date [15].

Research into cell-based therapy for the treatment of focal cartilage defects started at the end of the last century, with several of these products already on the market in Europe and the United States [16,17]. Compared with microfracture, autologous chondrocyte implantation (ACI) resulted in better structural regeneration with minimal subchondral bone alterations, hence producing greater improvement in clinical outcomes [18]. However, ACI is a two-stage process that is costly, associated with long rehabilitation, and has variable surgical risk factors affecting success rate. Arthroscopically-assisted osteochondral autologous transplantation surgery (OATS) has been successful, with a cohort of nine patients achieving an average post-operative AOFAS Ankle-Hindfoot score of 80.2 [19]. Mosaicplasty, which involves harvesting cylindrical osteochondral grafts of different sizes for transplant into focal cartilage lesions, has achieved good preliminary results and patient satisfaction [20]. However, these procedures rely on harvesting healthy tissue from the same or other joints. Studies have reported donor-site morbidity from the knee after an OATS was performed to treat talar osteochondral defects, with patients having knee pain and patellar instability [21,22]. A systematic review and meta-analysis reported a 7.8% donor-site morbidity rate from the knee after OATS for capitellar osteochondritis dissecans [23]. Furthermore, the technique involves chondrocyte dedifferentiation [24] and, ultimately, fails to reproduce the hyaline characteristics of original articular cartilage [25].

Mesenchymal stem cells (MSCs) are multipotent adult stromal cells that have the potential for self-renewal and multi-lineage differentiation. They can be isolated from various tissues such as bone marrow, adipose tissue, umbilical cord, dental pulp [26] and even from discarded fragments during surgery such as the infrapatellar fat pad and synovial membrane [27]. Depending on their tissue source, their methylation status determines their differentiation potentials. For example, the epigenetic memory of bone or adipose tissue-derived MSCs (AMSCs) favoured differentiation along an osteoblastic or adipocytic lineage [28]. MSCs are also immunomodulatory and can act in a paracrine fashion to aid tissue repair and promote a host of cellular responses, ranging from survival to proliferation and migration [29]. Linero et al. showed that AMSCs mediate bone regeneration mainly by releasing paracrine factors [30]. MSC paracrine signalling can facilitate chemotaxis of macrophages and endothelial lineage cells [31], and MSCs themselves secrete mitogens that stimulate the proliferation of keratinocytes and endothelial cells [29]. MSCs have, hence, successfully been used to treat chronic wounds and restart stalled healing processes [32]. Despite endogenous MSCs in synovial fluid being able to proliferate following tissue injury, numbers available for cartilage repair are few [33].

MSCs avoid the ethical dilemma surrounding the use of embryonic stem cells (ESCs). However, they can influence tumour development and lead to cancer drug resistance [34], for example, by increased VEGF expression, leading to angiogenesis in pancreatic carcinoma [35]. Furthermore, when integrated into tumour-associated stroma, they can cause cancer cells to increase their metastatic potency [36]. MSCs can become immunogenic in certain situations, for example, after IFNγ stimulation, umbilical cord-derived MSCs increase MHC class I and develop MHC class II expression [37].

Given the increasing interest in MSCs for treating focal cartilage defects, many clinical studies have been performed to assess their efficacy in terms of functional and patient-reported outcomes, especially in relation to the site of MSCs extraction. While the conventional site of harvest of MSCs is the bone marrow, AMSC is an increasingly popular choice due to ease of harvest [38], purported higher proliferative capacity [39] and anti-inflammatory properties [40]. Aside from being administered as a culture-expanded population of MSCs, AMSCs can also be administered in the form of Stromal Vascular Fraction (SVF), a mixture of heterogeneous cells containing a fraction of AMSCs as well as pericytes, endothelial cells, smooth muscle cells, fibroblasts, etc. [41]. Although SVF contains a fraction of AMSCs, since they express different cell markers and possesses different properties, they are not strictly defined as MSCs [42].

This systematic review and meta-analysis aims to review the potential of AMSCs and other AMSC-based therapies such as SVF for cartilage repair in vivo.

## 2. Methods

### 2.1. Search Algorithm

This systematic review was conducted in accordance with Preferred Reporting Items for Systematic Reviews and Meta-Analysis (PRISMA) guidelines [43]. A comprehensive literature search from conception to July 2021 was conducted using the following databases: (1) Ovid MEDLINE(R) (1946 to Present with Daily and Weekly Update, Epub Ahead of Print, In-Process & Other Non-Indexed Citations), (2) Ovid Embase (1910 to Present), (3) SCOPUS, (4) Web of Science and (5) Cochrane Library. References of included studies will be searched as well as studies that cited any of the included studies. Equivalent combinations of the following search terms were used: “adipose tissue-derived” AND “mesenchymal stem cells” AND “cartilage regeneration”. A detailed search strategy is available in Table S1. This review was prospectively registered in the International Prospective Register of Systematic Reviews PROSPERO (registration number: CRD42021267382, available from: https://www.crd.york.ac.uk/PROSPERO/display_record.php?RecordID=267382, accessed on 13 August 2021).

Title and abstract screening were independently performed by HM and VL, followed by full-text screening. A third reviewer (WK) was contacted for any unresolvable disagreements. A “snowball search” was performed whereby references of included studies, as well as studies that cited any of the included studies were independently searched by HM and VL.

### 2.2. Inclusion and Exclusion Criteria

Inclusion and exclusion criteria was created using the PICOS model (Population, Intervention, Comparison, Outcome, Study type) [44]. Detailed criteria are shown in Table 1.

### 2.3. Data Extraction

Data extraction was independently performed by HM and VL, with a third reviewer (WK) to resolve disagreements. Data were extracted into data tables created in a standardised excel spreadsheet for evidence synthesis and risk of bias analysis. Data from each study were split into five categories:

1. Study characteristics, such as study design, level of evidence, outcome measures and duration of follow-up.
2. Subject information such as subject model, mean age, mean BMI, percentage of female subjects and ethnicity.
3. Intervention information, including method of AMSC administration, AMSC cell count.
4. Results, including any complications arising during follow-up.
5. Outcome measures that were pertinent to cartilage regeneration would also be extracted in detail. This would include clinical scores related to cartilage damage, imaging scores and histological cartilage repair scores (Numerical data were extracted corrected to 3 significant figures).

### 2.4. Data Analysis

Comparable quantitative measures were selected for data extraction and meta-analysis. In terms of clinical scores, this includes the Visual Analogue Scale (VAS), a psychometric scale that measures the amount of pain a patient feels across a continuum [45]. We have presented VAS scores from 0 to 10 where 10 represents the most pain. Data pertaining to knee injury and Osteoarthritis Outcome Score (KOOS), which is designed to evaluate the short- and long-term symptoms and function in patients with knee injury that can result in post-traumatic OA or in primary OA [46], were also included. We also included the KOOS score of its five different subgroups (1) Knee-related symptoms (Symptoms); (2) Pain; (3) Function in daily living (ADL); (4) Function in Sports and Recreation (Sport & Rec); (5) and Knee-related quality of Life (QoL). Scores are presented on a 0 to 100 scale where 0 represents knee problems and 100 represents no knee problems. Western Ontario and McMaster Universities Osteoarthritis Index (WOMAC) scores were also selected for data extraction and analysis. WOMAC encompasses three main groups including pain, stiffness and function domains and was designed to evaluate OA specifically. WOMAC is expressed on a 0 to 100 scale where 100 means the worst pain, stiffness and physical function [47].

Regarding imaging outcomes, Magnetic Resonance Observation of Cartilage Repair Tissue (MOCART scores) were also selected for meta-analysis since it is a comparable quantitative outcome measure used in multiple studies [48]. MOCART score is an MRI score used for post-operative assessment of repaired cartilage in regards to its adjacent native hyaline cartilage in terms of morphology and signal intensity. It comprises of nine pertinent variables, including (1) the degree of filling of the defect; (2) the integration to the border zone; (3) the description of the surface; (4) description of the structure; (5) the signal intensity; (6) the status of the subchondral lamina; (7) the status of the subchondral bone; (8) the appearance of adhesions; (9) the presence of synovitis. This allows for the quantification of post-operative morphological MRI results and evaluation of repair, comparing the repaired tissue with adjacent cartilage [49]. Final MOCART scores of studies were extracted as the studies conducted MOCART scoring at different time points.

Forest plots were conducted using RStudio (metafor package [50]) with the extracted VAS, KOOS, WOMAC and MOCART results. We provided each subgroup with Cochran’s Q and the I^2^ statistic as measures of heterogeneity to account for the variability in effect size and proportion of variance attributable to study heterogeneity, p-values for heterogeneity were shown in forest plots, while *p*-values for overall effects were reported in text. A random effects model is adopted in the assessment of the aforementioned scores since the results are heterogenous (I^2^ ≥ 50).

Qualitative results or other results that were not selected for meta-analysis were extracted and presented in a table, as well as discussed in depth in the text. These were divided into clinical outcomes, imaging outcomes, histological outcomes and others.

### 2.5. Assessing Risk of Bias

Quality assessment was carried out independently by HM and VL using Cochrane’s RoB 2.0 tool for randomized trials, and Cochrane’s ROBINS-I tool for non-randomized trials. Disagreements were consulted with a third reviewer (WK).

Cochrane’s RoB 2.0 tool comprises five domains: (1) randomisation process; (2) deviations from intended interventions; (3) missing outcome data; (4) measurement of the outcome; (5) selection of the reported results [51]. Each domain was assessed as low risk, some concerns or high risk. We classified the overall risk of bias of each randomized study according to the RoB protocol.

Cochrane’s ROBINS-I tool comprises of seven domains: (1) bias due to confounding; (2) in selection of participants into the study; (3) in classification of interventions; (4) due to deviations from intended deviations; (5) due to missing data; (6) in measurement of outcomes; (7) in selection of reported result [52]. Each domain was assessed as Low, Moderate, Serious and Critical risk of bias. The summary of the results of both RoB and ROBINS-I were presented in a graph showing overall results stratified by their respective domains.

Data visualisation was conducted using RStudio (robvis package [53]).

## 3. Results

### 3.1. Search Results

**Figure 1:**
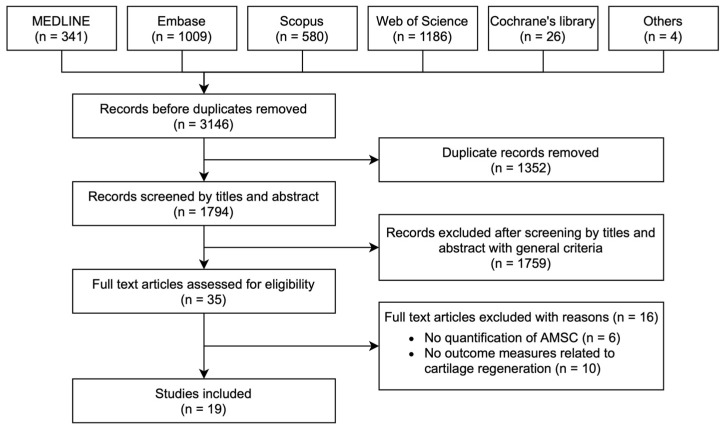
Flow diagram showing screening and selection process of studies.

We identified 3146 records after our literature search across the MEDLINE, EMBASE, Scopus, Web of Science and the Cochrane library, with 1794 records left after removal of duplicates (Figure 1). After screening by titles and abstracts with general criteria, 35 records were selected for full-text review. Nineteen records were selected for qualitative analysis after application of selection criteria.

### 3.2. Characteristics of Selected Studies

Details of study design and intervention are summarized in Table 2. There are six randomized trials and 10 non-randomized trials, including retrospective and prospective cohort studies as well as case series, four were dose-escalating studies. Two studies were on the same cohort of patients and were treated as a single trial [54,55]. Regarding the intervention, two studies utilized AMSCs from the infrapatellar fat pad, while other studies used adipose tissue of diverse origins including from the abdominal and gluteal region. Fifteen studies administered AMSCs via injection, while three studies administered AMSC by implantation. Three studies administered AMSC-based therapy twice [56,57,58]. Five studies used stromal vascular fraction as opposed to culture-expanded AMSCs. In eight studies, AMSC administration was also concurrent with other therapies, such as microfracture, arthroscopic debridement or injection of platelet-rich plasma (PRP) [56,57,59,60,61,62,63]. Cell count of AMSCs ranges from 10^6^ to 10^8^. Participant details of the included studies can be seen in Table 3. Three studies included patients with cartilage defects of the knee for a total of 73 patients, while 15 studies included patients with knee osteoarthritis for a total of 368. Outcome details and results of the included studies are summarized in Table 4. The duration of follow-up ranged from 6 to 36 months. Studies evaluated outcome measures in three major categories, clinical outcomes, imaging outcomes and histological outcomes.

### 3.3. Administration of AMSCs Leads to Improvement of Cartilage Defects

#### 3.3.1. Improvement of Clinical Outcomes

The majority of studies reported an improvement in clinical scores assessed using different scoring systems after the administration of AMSCs. In terms of pain symptoms, nine studies reported an improvement as assessed by VAS [54,55,58,59,61,64,65,66,69,72] and two studies reported improvement assessed by the Numeric Pain Rating Scale (NPRS) [56,57]. Two studies showed an improvement in overall patient health as assessed by the 36-Item Short Form Survey (SF-36) [58,70].

AMSCs were able to improve clinical outcomes as assessed by knee health indices, eight studies reported an improvement in KOOS [54,55,56,57,63,64,66,67,68], two studies reported an improvement in their Knee Society Score (KSS) [54,55,69], three studies reported an improvement in the International Knee Documentation Committee (IKDC) scores [60,62,64] and a further four studies reported an increase in knee health as assessed by the Tegner Activity Scale and Lysholm Knee Questionnaire [60,61,62,69]. One study reported an improvement in the Hospital for Special Surgery knee score (HSS-KS) [69] and one other study reported an improvement in Range of Motion testing [59].

For Osteoarthritis specific evaluation, 11 studies utilized the WOMAC scale and assessed the condition of osteoarthritic patients, all of which reported improvement after AMSC-based therapy.

**Figure 2:**
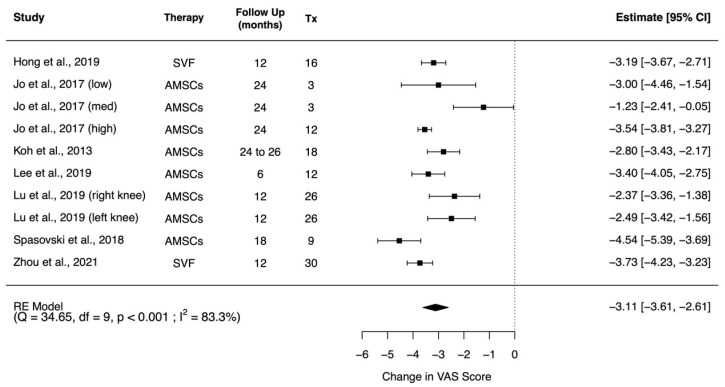
Forest plot on the administration of AMSC-based therapy and its effect in improving pain in VAS score. (Abbreviations: AMSC, Adipose tissue-derived mesenchymal stem cells; Tx, sample size of treated subjects; CI, Confidence Intervals; VAS, Visual Analogue Scale).

Quantitative analyses also corroborate the aforementioned results. Seven studies reported VAS in a comparable manner [54,58,59,61,66,69,72]. The study of Jo et al. was a dose-escalating study and the results are included as different entries [54,55]. In Lu et al., bilateral osteoarthritis patients were included and the results were reported as left and right knee cohorts, these were included separately [58]. A random effects meta-analysis revealed a significant improvement in VAS scores at end point (mean difference: −3.30; 95% CI (−3.72, −2.89), *p* < 0.001), comparing the baseline of the same cohort of participants (Figure 2).

**Figure 3:**
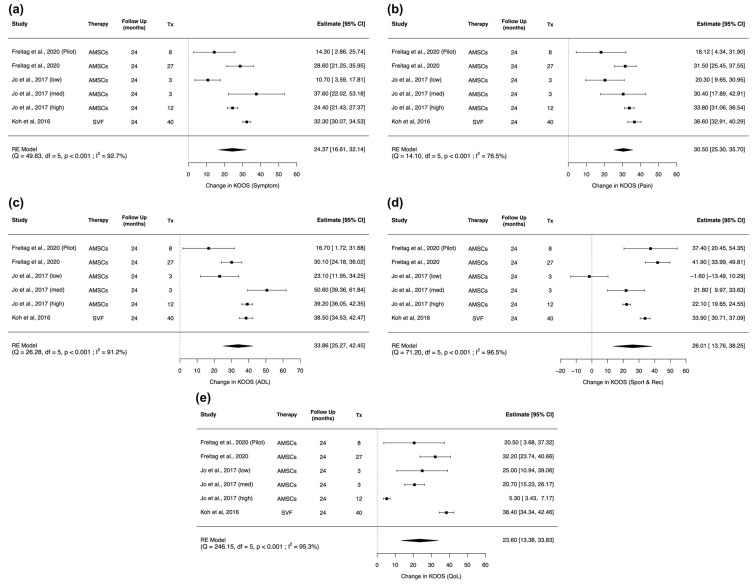
Forest plot on the administration of AMSC-based therapy and its effect in improving KOOS scores in terms of its five subgroups including: (**a**) Symptom; (**b**) Pain; (**c**) ADL; (**d**) Sports and Rec; (**e**) QoL. (Abbreviations: AMSC, Adipose tissue-derived mesenchymal stem cells; Tx, sample size of treated subjects; CI, Confidence Intervals; KOOS, Knee injury and Osteoarthritis Outcome Score; Symptoms, other symptom; ADL, function in daily living; Sports & Rec, function in sport and recreation; QoL, knee-related Quality of life).

Four studies reported comparable KOOS scores, and random effects meta-analysis was conducted on each of the KOOS subgroup results [54,56,57,63]. Again, Jo et al. was a dose-escalating study and its three cohorts (low dose, medium dose, high dose) were included separately [54,55]. Pooled analysis shows that there was a significant improvement in all five KOOS subgroups (Figure 3). These include KOOS Symptoms (mean difference: −24.37; 95% CI (16.61, 32.14), *p* < 0.001) (Figure 3a); Pain (mean difference: 30.50; 95% CI (25.30, 35.70), *p* < 0.001) (Figure 3b); ADL (mean difference: 33.86; 95% CI (25.27, 42.45), *p* < 0.001) (Figure 3c); Sports & Rec (mean difference: 26.01; 95% CI (13.76, 38.25), *p* < 0.001) (Figure 3d); QoL (mean difference: −23.60; 95% CI (13.38, 33.83), *p* < 0.001) (Figure 3e). Although the overall results are still significant, the low dose cohort in Jo et al. shows no improvement in KOOS Sports & Rec scores after AMSC administration.

**Figure 4:**
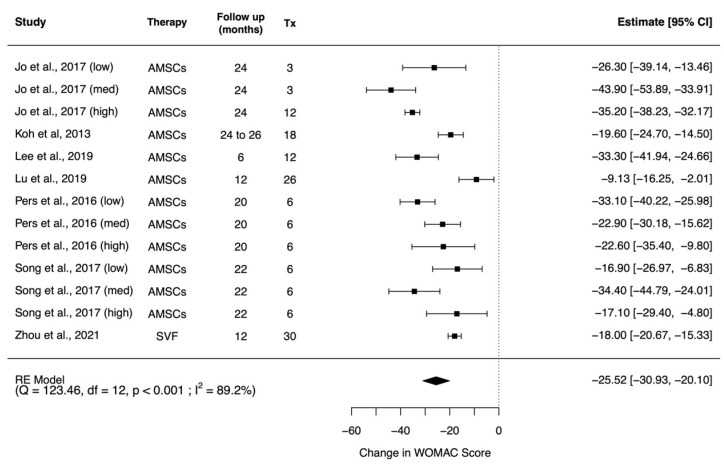
Forest plot on the administration of AMSC-based therapy and its effect in improving WOMAC scores. (Abbreviations: AMSC, Adipose tissue-derived mesenchymal stem cells; Tx, sample size of treated subjects; CI, Confidence Intervals; WOMAC, Western Ontario and McMaster Universities Osteoarthritis Index).

Six studies reported WOMAC results in a comparable method and were eligible for quantitative analysis [54,58,61,66,67,70,72]. Three studies with WOMAC results were dose-escalating studies and were again included in the meta-analysis separately respective to their dosage [54,67,70]. Random effects meta-analysis of the results revealed that after administration of AMSCs, a significant improvement at the end point of the study was found in WOMAC results (mean difference: −25.52; 95% CI (−30.93, −20.10), *p* < 0.001) when compared with the baseline of the same cohort (Figure 4).

The combined results show that AMSCs are effective in improving clinical outcomes of focal cartilage defects, from pain symptoms to knee health scores as well as osteoarthritis symptoms.

#### 3.3.2. Improvement of Imaging Outcomes

AMSC amelioration of focal cartilage defects is further supported by the MRI imaging outcomes by 14 studies. From MRI investigations, three studies showed cartilage regeneration by increased cartilage volume or depth [58,68,70]. While one other study reported significantly decreased cartilage defect depth [54,55], another study reported non-significant cartilage defect change with AMSC administration compared with significant cartilage defect increase in patients without AMSC administration [66]. Standardized imaging assessment systems were also utilized. Two studies utilized the Whole-Organ Magnetic Resonance Imaging Score (WORMS), with one reporting significant improvement and the other reporting non-significant changes [59,71].

Cartilage quality and composition was also evaluated in an MRI investigation using either T1rho mapping, T2 cartilage mapping or with delayed gadolinium-enhanced MRI of cartilage (dGEMRIC) by four studies [56,57,67,71]. Results showed an increase in cartilage condition from improved glycosaminoglycan/proteoglycan content as well as cartilage maturation.

**Figure 5:**
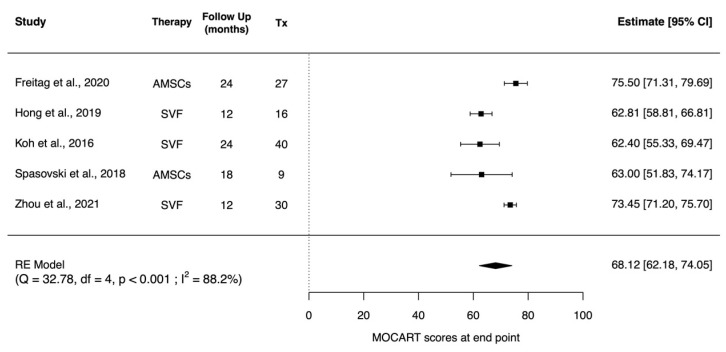
Forest plot showing MOCART scores at end point after AMSC administration. (Abbreviations: AMSC, Adipose tissue-derived mesenchymal stem cells; Tx, sample size of treated subjects; CI, Confidence Intervals; MOCART, Magnetic Resonance Observation of Cartilage Repair Tissue).

Seven studies evaluated cartilage regeneration with the MOCART score, with all studies showing significant progressive improvement or improvement when compared with control [56,57,59,63,64,69,72]. From the seven studies, five were comparable for random effects meta-analysis [57,59,63,69,72]. Pooled results of MOCART scores taken at the end point show the presence of cartilage regeneration (mean: 68.12; 95% CI (62.18, 74.05); *p* < 0.001) (Figure 5).

Two studies also conducted alternative imaging investigations in the form of sonography and plain radiography. Sonographic investigations showed improved clarity and integrity of the soft tissue-cartilage but no increase in cartilage depth [65], while plain radiography showed neither improvement nor further degradation of cartilage [69].

#### 3.3.3. Improvement of Histological Outcomes

Four studies evaluated cartilage regeneration histologically. Two studies evaluated using the International Cartilage Repair Society (ICRS) II histologic score. Three studies showed hyaline-like characteristics in the regenerated cartilage, such as the presence of safranin O and type II collagen in the regenerated cartilage [54,63,64]. One study found a stem cell graft-like sheet of stem cells [67].

#### 3.3.4. Other Improvements

One study evaluated the molecular profile of synovial fluid. The catabolic/anabolic profile was altered with decreased metalloproteinase 2 (MMP2) and increased insulin-like growth factor type 1 (IGF1). There was also a decrease in pro-inflammatory cytokines (IL1 β, IL6 and IL8) and an increase in anti-inflammatory cytokines (IL10) in the synovial fluid [65].

### 3.4. Assessment of Methodological Bias

**Figure 6:**
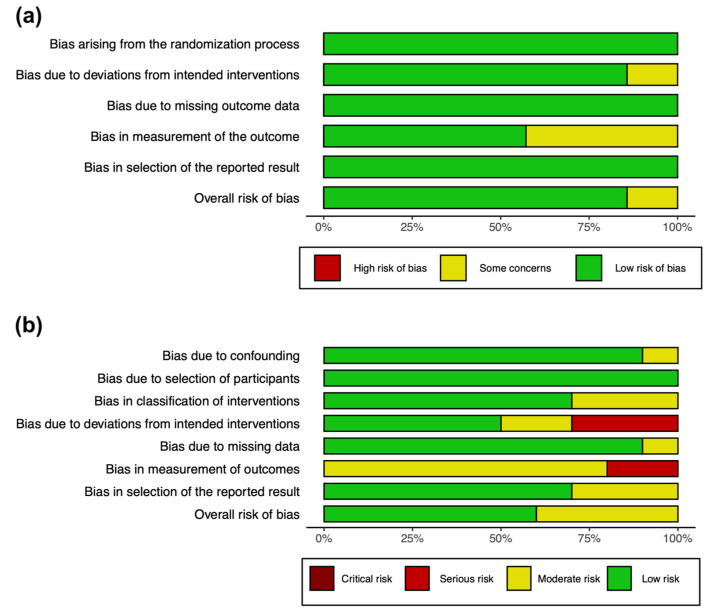
Summary graph showing overall risk of bias analysis using (**a**) RoB 2.0 tool in randomized studies and (**b**) ROBINS-I tool in non-randomized studies.

Overall, the risk of bias in the randomized studies was low (Figure 6a). The main source of bias was from the measurement of outcome, and this is due to outcome measures being measured and reported in the form of questionnaires.

For non-randomized studies, overall, there a is moderate risk of bias (Figure 6b). The major source of bias was from bias from the measurement of outcomes. As the participants were not blinded and the measurement outcomes were mostly questionnaires, there is a high risk of bias in terms of measurement. 

Detailed breakdown of the Risk of Bias analysis is available in Table S2.

## 4. Discussion

MSC-based therapies for cartilage defects induce hyaline-like cartilage regeneration and, therefore, have the potential to improve clinical outcomes in patients with cartilage defects. This disease-modifying approach is vastly different to conventional palliative therapies. Our primary findings show that in human studies, after the administration of AMSCs, clinical, histological or imaging outcome measures were improved, although there is variation in the type of study included, source and type of MSCs and method of administration. Included studies can be divided into randomized controlled trials and non-randomized trials, such as case series, retrospective studies and single cohort studies.

### 4.1. Optimal Source of Stem Cell

AMSCs are an increasingly attractive source for MSCs. A lot of progress on MSC-based cell therapy was achieved using bone marrow-derived MSCs (BMSCs), which was the dominant source of MSCs [73], however, the recent literature has expanded to including MSCs derived from almost all human tissue, including pluripotent stem cells. Among numerous options, adipose tissue has emerged as a dependable and rich source of MSCs, with regards to increased quantity, higher yield, lack of ethical issues and ease of harvest with minimally invasive procedures such as liposuction [74]. In vivo studies show that the differentiation potential of AMSCs is less attenuated by age when compared with BMSCs [75], and AMSCs have better immunosuppressive function [40]. AMSCs also have higher proliferation potential according to growth curve, cell cycle and telomerase activity analyses [39], although other studies have suggested that the differentiation potential of both types of MSCs are comparable [28,38,76]. Overall, while the exact properties of AMSCs compared with other MSCs have yet to be ascertained, AMSC-based therapies may be preferable to other MSC sources due to ease of harvest and having fewer ethical hurdles to overcome, which would aid its expansion in the healthcare market.

In our included studies, AMSCs were either derived from adipose tissue such as gluteal or abdominal adipose tissue or the infrapatellar fat pad (IFPFs). The ideal source for AMSCs is still a subject under research, however, IFPFs are an attractive source compared with adipose tissue since IFPF is usually obtained during the resection of inflamed tissue in knee arthroscopy [77]. IFPF-MSCs also display similar surface markers compared with other cells around the knee joint, and may reduce immunologic rejection [78]. Ding et al. suggested that IFPF-derived AMSCs may have higher proliferative potential than AMSCs derived from abdominal fat in vitro, although in vivo studies are needed to support this finding [78].

AMSCs were administered as culture-expanded AMSC or SVFs. Studies characterized the injected AMSCs by cell count. As mentioned previously, SVFs and AMSCs contain different minimal defining criteria. Research has shown that culture-expanded AMSCs result in better therapeutic potential owing to the larger number of MSCs as well as the higher potential to generate more trophic factors [79,80]. An in vivo study compared culture-expanded AMSCs with SVF regarding its use for osteoarthritis in sheep and reported better imaging, macroscopic and immunohistochemistry outcomes when AMSCs were used [81]. However, the culture expansion of MSCs resulted in lower migration and homing ability [82], and, therefore, culture-expanded AMSCs may require surgery to expose the site of lesion. Furthermore, SVFs that are minimally manipulated may be favoured for economic or regulatory reasons [83]. More comparative studies would be required to define the superior treatment for cartilage defects.

Among our included studies, Zhao et al. evaluated the use of allogeneic stem cells as opposed to autologous stem cells [71]. Allogeneic stem cells were obtained from three healthy donors and preclinical toxicity and chronic tumorigenicity were evaluated in vivo with no adverse events reported. Allogeneic MSC transplants have been previously demonstrated in animals as well as in human clinical trials, again with no adverse events reported [84,85]. This may be due to the low immunogenicity of MSCs as well as the immune-privileged character of cartilage tissue.

No severe adverse events were reported in our studies, and the safety of AMSC-based therapies has been previously reviewed in the literature, showing no permanent adverse effects [86]. Nevertheless, further research could evaluate the risks of harvesting adipose tissue for AMSC-based therapy via liposuction, investigate the interaction of AMSCs with the tumour microenvironment and evaluate the long-term safety of AMSC injection for cartilage defects with large sample size studies. The optimal source of adipose tissue for harvesting AMSCs is still under debate, and further studies are needed to ascertain the best form of AMSC-based therapies, i.e., culture-expanded AMSCs or SVFs, the use of allogeneic or autologous stem cells, as well as long-term safety.

### 4.2. Augmenting the Function of AMSCs

Despite the benefits of AMSCs, the outcomes of advanced clinical trials have sometimes fallen short of expectations [87]. This could be due to the vast dimensions across which MSC heterogeneity is present, such as among donors, tissue sources and even cell subpopulations with the same origin [88], and studies showing that MSCs disappeared after 24 h post-infusion [89]. It is, hence, prudent to ensure that studies utilising AMSCs adhere to well-defined international guidelines such as the International Federation for Adipose Therapeutics and Science (IFATS) [41] while pursuing novel avenues to artificially boost AMSC potency to overcome the shortcomings of naïve AMSCs.

AMSCs can be pre-conditioned in vitro, which prepares it for the harsh microenvironment of the host, enhances its migration to its site of action, or enhances its biological properties. Given that synovial inflammation plays a key role in the pathogenies of osteoarthritis, and is associated with cartilage destruction and pain, enhancing the immunomodulatory function of AMSCs has gained a lot of attention [90]. Pre-treating AMSCs with pro-inflammatory stimuli increases their immunosuppressive and anti-inflammatory potential by reducing NF-κB activity and promoting macrophage differentiation into the M2 anti-inflammatory phenotype [91]. Studies have shown that AMSCs express toll-like receptors (TLRs), which can be exploited via specific TLF-agonist engagement to affect their migration, secretion of immune modulating factors and induce a change in cell fate [92]. Furthermore, IFNγ-stimulated MSCs can enhance chondrogenesis [91]. However, the degree to which differentiation is affected depends on the MSC source, despite ligation of the same TLR, is under investigation [93], and future, in-depth comparative studies are needed to determine the best MSC source for chondrogenic differentiation in this scenario.

AMSCs are also amenable to genetic modification and may be adequate vehicles for gene delivery [94]. Targeted overexpression of microRNAs have produced favourable clinical outcomes [95,96]. miR-302 transfection increased proliferation and inhibited oxidant-induced cell death in AMSCs, and can be used to enhance the therapeutic efficacy of AMSCs in vivo [97]. However, studies have shown that genetic manipulation can affect differentiation potential, although results are conflicting and the effect on chondrogenic differentiation is unknown [98]. As described above, the immunomodulatory potential of AMSCs to promote cartilage repair is attractive, and genetic engineering has the potential to improve immunomodulation. CTLA4Ig-overexpressing AMSCs have been shown in a mouse model to protect against cartilage destruction and ameliorated severe rheumatoid arthritis [99]. Nevertheless, there is a risk of tumorigenicity and immunogenicity [100], and studies that examine the long-term clinical outcomes of using genetically modified AMSCs at a local and systemic level have yet to be published.

The niche microenvironment is crucial for stem cell integrity, fate and behaviour, and the regulation between an active and quiescent state [101]. Culture conditions must be optimised and can increase the biological potency of AMSCs. Conventional practice involves growing MSCs on a two-dimensional system as monolayers. This is artificial and lacks the key cell–cell and cell–extracellular matrix contact present in vivo. Three-dimensional cultured MSCs have shown superior expansive and differentiation potential, such as undergoing large-scale in vitro chondrogenic differentiation and enhanced in vivo cartilage formation in an animal model [102]. MSC aggregation into spheroids can also augment their immunomodulatory ability, as shown in a mouse model of cartilage damage [103]. Nevertheless, with the increased complexity of three-dimensional culture, it is necessary to regulate the time duration of the culture and size of spheroids formed [104]. Furthermore, studies have reported that AMSCs cultured under hypoxic (2% oxygen) conditions enhanced early chondrogenic differentiation, while decreasing osteogenesis, thus, favoured chondrocyte formation [105].

Finally, AMSC therapy can be combined with concomitant application after other bioactive molecules, or performed with other conventional procedures. Two studies injected AMSCs or SVF with PRP [61,68]. In vitro research has suggested that PRP optimizes MSC-based therapy by stimulating MSC proliferation, migration and immune modulation without affecting differentiation potential [106]. PRP may also optimize MSC-based therapy for cartilage regeneration by enhancing chondrogenic differentiation [107]. However these studies did not investigate the effect of PRP alone, making it hard to determine the actual role PRP has on chondrogenesis when administered together with AMSCs. Koh et al. compared AMSCs concurrent with microfracture therapy with just microfracture therapy [63] and reported improved radiologic and KOOS pain and function scores when AMSCs were administered together with microfracture. Nevertheless, only large cartilage defects (≥3 cm^2^) were investigated, and future research is needed to investigate the effects of AMSC and microfracture co-therapy for lesions of different sizes.

### 4.3. Methods of AMSC Administration

Methods of AMSC administration varied greatly in the included studies, though most studies favoured an intra-articular injection of AMSCs or SVF. Three studies investigated the use of implantation of AMSCs, none of which reported any adverse events [60,62,64]. Kim et al. compared implantation versus injection of AMSCs in their study and reported a significant difference [60], suggesting that in the implantation group, ICRS scores and clinical scores such as IKDC and Tegner scores were higher than those in the injection group. The article suggests that the cartilage regenerated by implantation has greater durability than that of injection. This may also be due to better cell retention and survival at the lesion site in implantation compared with injection.

Some studies also compared the use of AMSC therapy and other pre-existing therapies for focal cartilage defects. Hong et al. compared SVF with the injection of hyaluronic acid [59], while Lu et al. compared the AMSC injection against the injection of hyaluronic acid [58]. Both studies reported significant improvement in the AMSC or SVF group when compared with the control group with only the injection of hyaluronic acid. However, no study compared the efficacy of AMSC with SVF. Studies investigating cellular therapies for knee osteoarthritis suggested that iatrogenic complication may be higher in SVF groups, with more frequent knee effusion (SVF 8%, AMSC 2%) [108] and minor complications related to the fat harvest site (SVF 34%, AMSC 5%). However, clinical outcomes such as pain VAS improved earlier and to a greater degree in the AMSC group than VAS group.

Two included studies utilised arthroscopies prior to AMSC injection. Jo et al. only used arthroscopy to examine the patient and guide AMSC administration [54,55]. However, Zhao et al. performed arthroscopic debridement, which could lead to bias [71]. Arthroscopic debridement removes inflammatory synovial fluid, which impedes AMSC adhesion but also enhances the immunomodulatory profile of the AMSC secretome. Further research is needed to determine the effect of arthroscopy on the outcomes of AMSC treatment.

### 4.4. Improved Clinical Outcomes

All studies concluded that AMSC administration correlates with an improvement in pain and functional outcomes. Pooled analyses of WOMAC scores showed a statistically significant improvement in all studies across all follow-up times, regardless of the dose of SVF or AMSC used. This suggests that disease modification is long term, rather than simply acting as short-term analgesics, hence avoiding the need for the repeated administration of MSC therapy. However, a meta-analysis that directly compares SVF and AMSC treatments therapies could not be performed due to the low number of studies; furthermore, heterogeneity in the studies precluded any subgroup analysis of AMSC or SVF therapy.

Four studies segregated their cohort to receive AMSCs at different doses, namely low, medium and high. Three studies reported a dose-dependent effect on clinical outcomes, with the group receiving a high AMSC dose having the greatest reduction in cartilage defects and best clinical improvements [54,70,71]. Despite this suggesting a relationship between the number of AMSCs administered and therapeutic effect, Pers et al. found that only the group that received a low AMSC dose experienced significant improvements in pain levels and function compared to baseline [67]. This could be explained by the fact that significant synovial inflammation was present in the low dose cohort, and, as stated previously, MSCs can be primed by an inflammatory environment to exert their immunomodulatory effects [91]. This could lead to lower costs and the increased speed of AMSC harvesting, increasing their appeal in clinical practice. Due to the low number of studies investigating the outcomes of AMSC doses, subgroup analyses were not performed. Further research is needed on the dose-dependent relationship between AMSC therapy and long-term clinical outcomes, and, perhaps, studies that investigate the difference between one low dose and multiple low dose AMSC therapy, rather than giving one large bolus dose.

Joint pain and loss of functionality are the major symptoms of osteoarthritis. In the present study, VAS scores and other pain-related outcomes have been shown to improve after AMSC administration. There is an improvement in KOOS scores as well, which suggests an improvement in both symptoms and functions and also in the patient’s quality of life. Improvement in such clinical parameters suggests the therapeutic potential of using AMSCs over conventional therapies for focal cartilage defects. The therapeutic potential of MSCs on osteoarthritis have been previously described [109,110]. In the present study, AMSCs are also shown to improve cartilage regeneration as well as OA symptom scores. Despite macroscopic appearance scoring being predictive of histological scoring [111], more studies should be performed to evaluate the correlation between macroscopic and histological appearance with functional improvement.

Most studies did not stratify their patients based on the severity of osteoarthritis. Given that an inflammatory environment encourages AMSCs to bring out their immunomodulatory effects, they may be most effective during end stage osteoarthritis, given that inflammation levels are highest as the disease progresses. The current literature remains divided, with Nguyen et al. suggesting better efficacy in patients with less severe osteoarthritis [112], while Tran et al. reported greatly reduced WOMAC scores 24 months post-treatment in patients with Kellgren–Lawrence (K–L) grade 3 than K–L grade 2 [113].

Additionally, studies could have stratified patients based on age or BMI. Studies have shown that ageing negatively impacted AMSC proliferation and reduced its chondrogenic differentiation ability in favour of adipogenic differentiation [114]. Obesity has been shown to be a key risk factor for osteoarthritis, however, the biological role of adipose-derived inflammation on MSC efficacy have yet to be investigated [115].

### 4.5. Hyaline-Like Cartilage Regeneration

The underlying mechanism of AMSCs amelioration of cartilage defects lie in the potential of AMSCs to generate hyaline-like cartilage. 

Previous in vitro studies have exhibited the chondrogenic differentiation ability of AMSCs with successful differentiation indicated by the immunohistological staining of type II collagen or expression of glycosaminoglycan in the regenerated tissue similar to hyaline cartilage [116,117,118]. Animal studies have also corroborated with in vitro results and evaluated the safety of the administration of MSCs [109,119]. Bone marrow-derived MSCs and synovium-derived MSCs have been shown to induce hyaline-like cartilage regeneration confirmed by biochemical results such as improved GAG content and histological results related to type II collagen expression and integration. Early human AMSC studies, such as Pak et al., showed probable cartilage regeneration in the knee joint after AMSC administration [120]. In our included studies, histological and imaging outcomes as well as biomarker analysis shows the presence of hyaline-like cartilage regeneration. Koh et al. performed histological staining and showed that participants receiving AMSC administration in concurrence with microfracture exhibited a higher degree of staining for safranin O and type II collagen than patients who received microfracture alone, suggesting the presence of hyaline cartilage regeneration after AMSC administration [63]. For imaging analysis, the MOCART tool assesses regenerated cartilage compared to its similarity in terms of morphology and signal intensity with the surrounding native hyaline cartilage. The improved MOCART score post AMSC administration therefore suggests the presence of hyaline-like properties of the regenerated cartilage. Aside from MOCART, Freitag et al. utilized additional T2-weighted mapping to evaluate the quality of the regenerated cartilage and showed progressive cartilage maturation over time [56,57]. These results point towards the ability of AMSCs to generate hyaline-like cartilage in humans.

Nevertheless, with an average follow-up time of 18.3 months, the long-term effects of AMSC therapy on cartilage repair are unknown. Jo et al. reported that cartilage degeneration occurred after two years post-treatment, perhaps due to desensitisation of the knee joint to AMSC therapy, or simply the need for another AMSC injection [54]. Park et al. provided more optimistic results, with all cartilage regeneration parameters remaining stable over a seven year follow-up period [121]. There is a need in the literature for more studies with long follow-up times to determine the long-lasting effects of AMSC therapy.

### 4.6. Strengths and Limitations

Our study has several strengths, including an extensive search strategy, robust inclusion and exclusion criteria, and thorough data extraction in the form of both quantitative measures and qualitative outcomes, with meta-analysis of quantitative outcomes. However, the included studies in the present review show a high degree of heterogeneity from different methods of administration, harvesting, type of MSC-based therapy, duration of follow-up as well as the disease state of participants. Furthermore, the different methods of reporting mean that we were only able to conduct meta-analyses on quantifiable outcomes reported in a comparable manner, creating a bias towards studies with similar quantifiable outcomes. WOMAC and VAS are non-specific scoring systems, and it may be better to create a novel patient-reported outcome measure specifically for patients treated with MSC-based therapies. Risk of bias analysis also shows a high risk of measurement bias due to most of the clinical outcome measures being in the form of self-reported questionnaires. There is an especially high risk of bias in the measurement of the outcomes in non-randomized and non-blinded studies.

## 5. Conclusions

MSC-based therapies hold high potential for restorative treatment of cartilage defects, and, in recent years, there has been an interest in the use of AMSCs due to their ease of collection and abundance. This systematic review and meta-analysis compiled the findings in human studies for the administration of AMSC-based therapies for amelioration of cartilage defects. The evidence suggests that there is an improvement in clinical, imaging and histological outcomes after AMSC or SVF administration with no severe adverse events reported. Despite heterogeneity in studies included, there is evidence supporting the use of AMSCs or SVFs for focal cartilage defects. We recommend researchers establish the roles of biochemical components that stimulate cartilage repair after AMSC therapy, as well as making chondrocyte gene expression, cartilage macroscopic appearance and histological scores important outcome measures, in addition to functional and clinical outcomes. Establishing the most efficient and safest method for MSC extraction, culture and delivery, and performing studies with long follow-up times to determine the lasting implications should enable future high-quality research to provide the evidence needed to bring AMSC-based therapies into the market to tackle major public health challenges such as OA.

## Figures and Tables

**Table 1:** PICOS inclusion and exclusion criteria for study selection.

Domain	Inclusion Criteria	Exclusion Criteria
**Population**	Clinical study conducted on human populations regardless of age, gender, ethnicity, demography, geography, presenting complaints or history with cartilage damage	Studies conducted on animals. Ex vivo studies and in vitro studies
**Intervention**	Studies that evaluate the use of adipose tissue-derived mesenchymal stem cell transplant as a method to induce cartilage regeneration	Studies which use AMSCs in an unquantifiable manner
**Comparison**	Adipose tissue-derived mesenchymal stem cells on cartilage regeneration that are compared with non-exposed control groups and or mesenchymal stem cells derived from other sources	Studies that treat cartilage loss not related to cartilage regeneration, such as for pain relief
**Outcome**	Any outcomes pertaining to the regeneration of cartilage will be included, such as macroscopic, radiological and histological scores	Studies where no outcome measures are directly related to cartilage regeneration
**Study Type**	English with full text available	Case reports

Abbreviations: AMSCs, adipose tissue-derived mesenchymal stem cells.

**Table 2:** Study design and intervention details of the included studies.

Author	Study Design	AMSC-Based Therapy	Administration Method	Control	Cell Count
Freitag et al., 2020 (Pilot) [56]	Pilot case series	Autologous abdominal AMSCs	Intra-articular injection at 1 week at 6 months (after arthroscopic abrasion arthroplasty)	N/A	5.21 × 10^6^
Freitag et al., 2020 [57]	Prospective case series	Autologous abdominal AMSCs	Intra-articular injection at 1 week at 6 months (after arthroscopic abrasion arthroplasty)	N/A	5.21 × 10^6^
Hong et al., 2019 [59]	Double blind randomized self-controlled trial	Autologous abdominal SVF	Intra-articular injection at cartilage lesion site (with arthroscopic debridement)	HA injection	SVF cell density (7.45 ± 3.73 × 10^6^/mL)
Jo et al., 2014 [55], 2017 [54]	Dose-escalation Cohort study	Autologous abdominal AMSCs	Intra-articular injection	Comparison between high, med, low dose	Low: 1.00 × 10^7^ Medium: 5.00 × 10^7^ High: 1.00 × 10^8^
Kim et al., 2015 [60]	Single cohort	Autologous gluteal AMSCs	Implantation onto fibrin glue scaffold over lesion site (with debridement)	N/A	4.01 × 10^6^
Koh et al., 2013 [61]	Therapeutic case series	Autologous infrapatellar fat pad AMSCs	Intra-articular injection (with PRP)	N/A	1.18 × 10^6^
Koh et al., 2014 [62]	Retrospective Case Series	Autologous gluteal AMSCs	Implantation (with debridement)	N/A	3.80 × 10^6^
Koh et al., 2016 [63]	Randomized prospective comparative study	Autologous gluteal SVF	Intra-articular injection (with microfracture)	Microfracture	4.97 × 10^6^
Kyriakidis et al., 2020 [64]	Case series	Autologous hypogastric derived AMSCs	Implantation onto three-dimensional matrix	N/A	Cells identified as AMSCs but not counted
Lapuente et al., 2020 [65]	Retrospective non-controlled study	Autologous abdominal SVF	Intra-articular injection	N/A	3.21 × 10^6^
Lee et al., 2019 [66]	Randomized double blind placebo controlled trial	Autologous abdominal AMSCs	Intra-articular injection	Normal saline injection	1.00 × 10^8^
Lu et al., 2019 [58]	Randomized double blind active-controlled controlled trial	Autologous abdominal AMSCs	Intra-articular injection at week 0 and 3	HA injection	5.00 × 10^7^
Pers et al., 2016 [67]	Single-arm, open-label, dose-escalating	Autologous AMSCs	Intra-articular injection	N/A	Low: 2.00 × 10^6^ Medium: 1.00 × 10^7^ High: 5.00 × 10^7^
Simunec et al., 2020 [68]	Comparative case series	Autologous SVF	Intra-articular injection (with PRP)	N/A	4.24–10.2 × 10^6^
Spasovski et al., 2018 [69]	Single-arm, open-label	Autologous abdominal AMSCs	Intra-articular injection	N/A	0.500–1.00 × 10^7^
Song et al., 2018 [70]	Randomized double-blinded dose-escalating Phase I trial with Open Phase IIa trial	Autologous AMSCs	Intra-articular injection	N/A	Low: 1.00 × 10^7^ Medium: 2.00 × 10^7^ High: 5.00 × 10^7^
Zhao et al., 2019 [71]	Randomized double blind placebo controlled trial	Allogeneic abdominal AMSCs	Intra-articular injection	Comparison between high, med, low dose	Low: 1.00 × 10^7^ Medium: 2.00 × 10^7^ High: 5.00 × 10^7^
Zhou et al., 2021 [72]	Randomized double blind placebo controlled trial	Autologous infrapatellar fat pad SVF	Intra-articular injection	Knee arthroscopic therapy	3.91 × 10^6^

Abbreviations: AMSCs, Adipose tissue-derived mesenchymal stem cells; SVF, Stromal Vascular Fraction; PRP, Platelet-rich Plasma.

**Table 3:** Participant details of the included studies.

Author	Model	Subjects	Control	Mean Age of Subjects	Mean Age of Control	BMI of Subject	BMI of Control	Sex Ratio of Subject (M–F)	Sex Ratio of Control (M–F)	Participant Ethnicity
Freitag et al., 2020 (Pilot) [56]	Patients with a focal full thickness chondral defect of the knee	8	N/A	23–52	N/A	25.2	N/A	4:4	N/A	Australian
Freitag et al., 2020 [57]	Knee OA patients (K–L grade IV)	27	N/A	53.6 ± 6.7	N/A	27.7 ± 5.3	N/A	18:9	N/A	Australian
Hong et al., 2019 [59]	Bilateral Knee OA patients (K–L grade II–III)	16	16 (16 knee)	52 ± 8.4	N/A	26.4 ± 1.8	N/A	3:13	N/A	Chinese
Jo et al., 2014 [55], 2017 [54]	Knee OA patients	18 (divided into low, medium, high dose)	N/A	N/A	N/A	N/A	N/A	N/A	N/A	Korean
Kim et al., 2015 [60]	Knee OA patients (K–L grade I–II)	Injection: 20Implantation: 20	N/A	59.3 ± 3.3	N/A	26.8 ± 2.4	N/A	14:26	N/A	Korean
Koh et al., 2013 [61]	Knee OA patients (K–L grade III–IV)	18	N/A	N/A	N/A	N/A	N/A	6:12	N/A	Korean
Koh et al., 2014 [62]	Knee OA patients (K–L grade I–II)	56 (60 knees)	N/A	56.6 ± 5.2	N/A	26.5 ± 3.0	N/A	22:34	N/A	Korean
Koh et al., 2016 [63]	Patients with ICRS grade III–IV cartilage defect on femoral condyle	40	40	38.4 ± 6.4	39.1 ± 7.1	26.8 ± 3.5	27.1 ± 3.1	14:26	16:24	Korean
Kyriakidis et al., 2020 [64]	Patients with ICRS grade III–IV cartilage defects of the knee	25	N/A	30.5	N/A	23.6	N/A	10:15	N/A	Greek
Lapuente et al., 2020 [65]	Knee OA patients (K–L grade III–IV)	50	N/A	50 to 89	N/A	N/A	N/A	N/A	N/A	Spanish
Lee et al., 2019 [66]	Knee OA patients (K–L grade II–IV)	12	12	62.2-± 6.5	63.2 ± 4.2	25.3 ± 4.9	25.4 ± 3.0	3:9	3:9	Korean
Lu et al., 2019 [58]	Knee OA patients (K–L grade I–III)	26	26	55.0 ± 9.2	59.6 ± 6.0	24.3 ± 3.0	24.3 ± 2.6	3:23	3:23	Chinese
Pers et al., 2016 [67]	Patients with Knee OA	18 (divided into low, medium, high dose)	N/A	64.6 ± 4.8	N/A	27.6 ± 5.6	N/A	8:10	N/A	French and German
Simunec et al., 2020 [68]	Knee OA patients (K–L grade III–IV)	12	N/A	61.0	N/A	26.4	N/A	7:5	N/A	German
Spasovski et al., 2018 [69]	Knee OA patients	9	N/A	63.0 ± 10.4	N/A	29.5 ± 4.0	N/A	3:6	N/A	Serbian
Song et al., 2018 [70]	Knee OA patients (K–L grade >II)	18 (divided into low, medium, high dose)	N/A	N/A	N/A	N/A	N/A	N/A	N/A	Chinese
Zhao et al., 2019 [71]	Knee OA patients	18 (divided into low, medium, high dose)	N/A	54.8 ± 10.2	N/A	24.5 ± 2.1	N/A	5:13	N/A	Chinese
Zhou et al., 2021 [72]	Knee OA patients	30	30	52.3 ± 1.2	51.8 ± 7.6	24.2 ± 2.5	23.6 ± 3.0	5:25	8:22	Chinese

Abbreviations: OA, Osteoarthritis; ICRS, International Cartilage Repair Society; K–L, Kellgren and Lawrence; (M–F), Male to Female ratio; BMI, Body Mass Index.

**Table 4:** Outcome details of the included studies.

Author	Outcome Measures	Follow-Up (Months)	Main Results	Complications	Level of Evidence
Freitag et al., 2020 (Pilot) [56]	WOMAC, KOOS, NPRS, MRI (ICRS score, MOCART score, T2 cartilage mapping)	24	Improvement in pain and function observed, corresponding with MRI imaging analysis showing cartilage regeneration	No serious adverse event occurred	IV
Freitag et al., 2020 [57]	WOMAC, KOOS, NPRS, Patient global impression of change; MRI (MOCART, T2 cartilage mapping)	24	Significant improvements in pain and function were observed, as well as hyaline-like cartilage regeneration	Treatment tolerated with no serious adverse events	IV
Hong et al., 2019 [59]	WOMAC, VAS, ROM, WORMS, MRI (MOCART)	12	VAS, WOMAC and ROM improved in patients with injection of SVF, MOCART revealed improvement of articular improvement compared with hyaluronic acid-treated knees	No severe adverse events observed	II
Jo et al., 2014 [55], 2017 [54]	WOMAC, KOOS, VAS, KSS, Knee pain, MRI (depth of cartilage)	24	Knee function improved (WOMAC, KSS, KOOS), reduced knee pain (VAS), Corresponding with MRI results, showing significant decreased cartilage defect depth at 2 years, as well as significant increased regenerated cartilage volume initially but non-significant from baseline after 2 years. Higher dose cohort has improved clinical outcomes compared with lower doses cohort.	No treatment-related adverse events	III
Kim et al., 2015 [60]	IKDC, Tegner, ICRS	24	Improved IKDC, Tegner activity scores, in both injection and implantation groups. Implantation group has better ICRS outcome compared with injection groups	N/A	III
Koh et al., 2013 [61]	WOMAC, VAS, Lysholm, MRI (cartilage whole-organ MRI score)	24–26	WOMAC, Lysholm, VAS scores improved. Radiography shows improved cartilage whole-organ MRI scores and was related to amount of stem cell injection	N/A	IV
Koh et al., 2014 [62]	IKDC, Tegner, ICRS	24–30	IKDC and Tegner scale scores were significantly improved as well as ICRS overall repair grades	No severe adverse events observed	IV
Koh et al., 2016 [63]	KOOS, symptom subscores, MOCART, ICRS histologic outcomes	24	MRI showed better cartilage coverage of lesion in patients receiving AMSCs with microfracture. Improvement in KOOS scores is significantly greater than in control	N/A	II
Kyriakidis et al., 2020 [64]	KOOS, VAS, IKDC, MRI (MOCART)	36	KOOS, IKDC subjective, Tegner activity, VAS for pain scores were improved. MRI findings showed filling of defect and integration to border zone in 65% of patients. Two patient underwent post-operative biopsies and histological analysis, showing presence of hyaline-tissue	No complications nor treatment-related adverse events were observed	IV
Lapuente et al., 2020 [65]	WOMAC, VAS, Synovial fluid profile, Articular ultrasound; Femoral articular cartilage sonographic evaluation (clarity, integrity, thickness in millimetre)	12	WOMAC, VAS scales improved, correlated with ultrasound observations. Biomarker analysis shows decrease in catabolic and pro-inflammatory pathways and increase in anabolic and anti-inflammatory pathways	No serious adverse event occurred	IV
Lee et al., 2019 [66]	WOMAC, KOOS, VAS, MRI (Modified Noyes grading system, with cartilage defect size)	6	WOMAC Score improvement at 6 months, MRI showed no significant change of defect size in injection group but increased defect in control	No serious adverse event occurred	II
Lu et al., 2019 [58]	WOMAC, VAS, SF-36, MRI (Knee cartilage volume)	12	More patients achieved 50% improvement in WOMAC and more increase in articular cartilage volume of both knees in the injection group than HA group as measured by MRI. Adverse events were comparable	No serious adverse event in injection group, mild moderates Adverse event. Serious Adverse Event of right knee joint infection in the HA group	II
Pers et al., 2016 [67]	WOMAC, KOOS, MRI (dGEMRIC), Histologic analysis, OARSI grading	20	WOMAC improved, dGEMRIC performed on 7 patients, which improved over time for 3 patients but opposite effect in the other 3 patients.	No serious adverse event occurred	IV
Simunec et al., 2020 [68]	KOOS, MRI	12	KOOS scores improved, MRI revealed widening of joint space, restructuring of cartilage and alleviation of effusions in treated joints	No serious adverse event occurred	IV
Spasovski et al., 2018 [69]	VAS, KSS, HSS-KS, Tegner-Lysholm, plain radiography, MRI (MOCART)	18	Improvement of KSS, HSS-KS, T-L scores, and significant improvement in MOCART score; Radiography shows neither improvement nor further joint degeneration	No serious adverse event occurred	IV
Song et al., 2018 [70]	WOMAC, NRS-11, SF-36, MRI (cartilage volume)	22 (96 weeks)	AMSCs improved pain, function and cartilage volume in the knee joint, with a dose-dependent effect	No serious adverse event occurred	IV
Zhao et al., 2019 [71]	WORMS, MRI	11 (48 weeks)	Improvement in WOMAC and SF-36 scores; Changes in T1rho, T2, T2star, R2star and ADC measurements suggests possible compositional changes in cartilage	No serious adverse event occurred	II
Zhou et al., 2021 [72]	WOMAC, VAS, MRI (MOCART)	12	WOMAC, VAS rest, VAS motion scores were found significantly lower than control group; MOCART scores of experiment groups were found significantly higher than experiment group; No significant difference in WOMAC stiffness scores	No serious adverse event occurred	II

Abbreviations: WOMAC, Western Ontario and McMaster Universities Osteoarthritis Index; VAS, Visual Analogue Scale; KOOS, Knee injury and Osteoarthritis Outcome Score; KSS, Knee Society Score; HSS-KS, Hospital for Special Surgery Knee Score; ROM, Range of Motion; NRS-11, Numeric Rating Scale-11; NPRS, Numeric Pain Rating Scale; SF-36, Short Form Health Survey-36; MRI, Magnetic Resonance Imaging; MOCART, Magnetic Resonance Observation of Cartilage Repair Tissue; WORMS, Whole-organ MRI score; dGEMRIC, delayed gadolinium-enhanced MRI of cartilage; ADC, Apparent diffusion coefficient; ICRS, International Cartilage Repair Society; IKDC, International Knee Documentation Committee.

## Data Availability

Data is contained within the article and supplementary material.

## Supplementary Materials

The following are available online at https://www.mdpi.com/article/10.3390/ph14121280/s1, Table S1: Detailed search strategy, Table S2A: Breakdown of risk of bias analysis using RoB 2.0 tool of randomized studies, Table S2B: Breakdown of risk of bias analysis using ROBINS-I tool of non-randomized studies Table S3: PRISMA checklist.
